# Antimicrobial and antiurease activities of newly synthesized morpholine derivatives containing an azole nucleus

**DOI:** 10.1007/s00044-012-0318-1

**Published:** 2012-11-29

**Authors:** Hakan Bektaş, Şule Ceylan, Neslihan Demirbaş, Şengül Alpay-Karaoğlu, Bahar Bilgin Sökmen

**Affiliations:** 1 Department of Chemistry, Faculty of Arts and Sciences, Giresun University, 28049 Giresun, Turkey; 2 Department of Forest Industry Engineering, Faculty of Forest, Artvin Coruh University, 08100 Artvin, Turkey; 3 Department of Chemistry, Faculty of Sciences, Karadeniz Technical University, 61080 Trabzon, Turkey; 4 Department of Biology, Faculty of Arts and Sciences, Rize University, 53100 Rize, Turkey

**Keywords:** Morpholine, 1,2,4-Triazole, 1,3,4-Oxadiazole, Mannich base, Antimicrobial activity, Antiurease activity

## Abstract

2-[6-(Morpholin-4-yl)pyridin-3-ylamino]acetohydrazide (**4**) was obtained starting from 6-morpholin-4-ylpyridin-3-amine (**2**) via the formation of ester (**3**) and then converted to the corresponding Schiff bases (**5, 6**) with the reaction with aromatic aldehydes. The carbothioamide (**9**), obtained from the reaction of hydrazide with phenylisothiocyanate, was converted to the corresponding 1,2,4-triazole (**11**) and 1,3,4-thiadiazole (**12**) derivatives by the treatment with NaOH or H_2_SO_4_, respectively. The cyclocondenzation of **9** with 4-chlorophenacyl bromide or ethyl bromoacetate produced the corresponding 1,3-thiazole (**10**) or 1,3-thiazolidine derivatives (**13**), respectively. Antimicrobial and antiurease activities of newly synthesized compounds were investigated. Some of them were found to be active on *M. smegmatis*, and they displayed activity toward *C. albicans* and *S. cerevisiae* in high concentration. Compound **10** proved to be the most potent showing an enzyme inhibition activity with an IC_50_ = 2.37 ± 0.19 μM.

## Introduction

Urea amidohydrolases (ureases) have been known as a class of large heteropolymeric enzymes with the active site containing two nickel (II) atoms and to accelerate hydrolysis of urea to ammonia gas with the reaction rate at least 10^14^ over the spontaneous reaction. Ureases are widely distributed in nature and are found in a variety of plants, algae, fungi, and bacteria (Kot *et al*., 2010). Medically, bacterial ureases have been reported as important virulence factors implicated in the pathogenesis of many clinical conditions such as pyelonephritis, hepatic coma, peptic ulceration, and the formation of injection-induced urinary stones and stomach cancer. The catalytic mechanism of their action has been believed to be the same of all urease inhibitors in which the amino acid sequences of the active site are principally conserved (Xiao *et al*., 2010). The active site of the native enzyme binds three water molecules and a hydroxide ion bridged between two nickel ions (Bachmeier *et al*., 2002). In the course of enzymatic reaction, urea replaces these three water molecules and bridges the two metal ions. The surrounding by a hydrogen-bonding network strongly activates the inert urea molecule; it is subsequently attacked by the hydroxide ion, forming a tetrahedral transition state. As a result, ammonia is released from the active site followed by the negatively charged carbamate (Adil *et al*., 2011). The latter decomposes rapidly and spontaneously, yielding a second molecule of ammonia. The ammonia generated may cause disruption to several metabolic functions in a large number of animal tissues and organs (Adil *et al*., 2011).

Urease is also indispensable for colonization of human gastric mucosa by *Helicobacter pylori*. The ammonia produced has been shown to be toxic for various gastric cell lines. Furthermore, urease activity was proposed to damage the gastric epithelium via its interaction with the immune system by stimulating an oxidative burst in human neutrophils (Ito *et al*., 1998). H_2_O_2_ generated in this oxidative burst probably reacts with ammonia and chloride to yield the toxic monochloramine (Kot *et al*., 2010). Finally, the ammonia may reach the serum and contribute to symptoms of hepatic encephalopathy in patients suffering from cirrhosis. Apart from ammonia, the carbon dioxide generated by urea hydrolysis may play a significant role for survival of *H. pylori* in the gastric mucosa (Cobena *et al*., 2008; Miroslawa *et al*., 2010; Xiao *et al*., 2010; Khan *et al*., 2010a, b; Ito *et al*., 1998; Keri *et al*., 2002; Ashiralieva and Kleiner, 2003).

Moreover, urea constitutes the predominant source of nitrogen containing fertilizers used in agriculture, accounting for 50 % of the total world fertilizer nitrogen consumption. However, the efficiency of urea is decreased by its hydrolysis with the enzyme urease to ammonia gas in soil. Besides the economic impact for farmers, NH_3_ lost to the atmosphere from applied urea causes eutrophication and acidification of natural ecosystems on a regional scale (Cobena *et al*., 2008).

Several classes of compounds have been reported as the agents having antiurease activity; among them hydroxamicacids are the best recognized urease inhibitors (Adil *et al*., 2011; Krajewska, 2009; Muri *et al.,* 2003). Phosphoramidates, another class of antiurease agents, have been reported as the most potent compounds (Amtul *et al.,* 2002; Kot *et al.,* 2001). However, the teratogenicity of hydroxamicacid in rats and degradation of phosphoramidates at low pH (Adil *et al*., 2011, Domínguez *et al*., 2008; Kreybig *et al*., 1968) restrict their use as a drug in vivo. Another class of compounds showing enzyme's inhibitory activity is polyphenols such as gallocatechin that is a polyphenol extracted from green tea and quercetin, a naturally occurring flavonoid having anti-*H. pylori* activity (Matsubara *et al*., 2003; Shin *et al*., 2005).

In addition, some 1,2,4-triazoles, 1,3,4-oxadiazoles, and 1,3,4-thiadiazoles have also been reported as the compounds possessing antiurease activity (Amtul *et al*., 2004; Aktay *et al*., 2009; Bekircan *et al*., 2008). Recently, some complexes of Schiff bases with metal ions showed significant inhibitory activities against urease (Shi *et al.,* 2007; You *et al.,* 2010) along with other metal complexes (Cheng *et al.,* 2009). However, owing to the presence of heavy metal atoms, these types of compounds can inflict toxic effects on human body (Duruibe *et al.,* 2007); hence, such molecules cannot be used as drugs.

During the recent decades, the human population being afflicted with life-threatening infectious diseases caused by multidrug-resistant Gram-positive and Gram-negative pathogen bacteria has been increasing at an alarming lscale around the world as a result of antimicrobial resistance. In spite of the wide range of antimicrobial drugs with different mechanisms of action used for the treatment of microbial infections either alone or in combination and also the existence of many compounds used in different phases of clinical trials, microbial infections have been posing a worldwide problem. There is already evidence that antimicrobial resistance is associated with an increase in mortality (Bayrak *et al*., 2010a, b, 2009a, b; Demirbas *et al*., 2009). The growing number of reports of antibiotic resistance worldwide has led to fears that some lethal human pathogens, such as *Mycobacterium tuberculosis*, will soon become untreatable (Dye and Williams, 2009; Dye and Phill, 2006; Koca *et al*., 2005; Zalavadiya *et al*., 2009). Tuberculosis (TB) causes the death of approximately three million patients in the world every year. These numbers make TB one of the leading infectious causes of death, eclipsed only by AIDS. Synthetic drugs for treating TB have been available for over half a century, but incidences of the disease continue to be on the rise worldwide. The causative organism, Mycobacterium tuberculosis, is a tremendously successful colonizer of the human host and is estimated to have latently infected approximately one-third of humanity. A growing number of immunocompromised patients are attributed to cancer chemotherapy, organ transplantation, and HIV infection, which are the major factors contributing to this increase. Therefore, it is necessary to search for and synthesize new classes of antimicrobial compounds that are effective against pathogenic microorganisms that have developed resistance to the antibiotics (Dye and Williams, 2009; Dye and Phill, 2006; Koca *et al*., 2005; Zalavadiya *et al*., 2009; Bayrak *et al*., 2010a, b).

In the field of medicinal chemistry, azoles belong to a class of antimicrobial agents that are widely used and studied because of their safety profile and high therapeutic index. Ribavirin, rizatriptan, alprazolam, vorozole, letrozole, and anastrozole are the best examples of drugs containing 1,2,4-triazole moiety (Ashok *et al*., 2007; Rao *et al*., 2006; Hancu *et al*., 2007; Cai *et al*., 2007). Among azole-based drugs, conazoles, such as itraconazole, fluconazole, voriconazole, and ravuconazole constitute a major class being used for the treatment of fungal infections (Yu *et al*., 2007; Gupta *et al*., 2007; Schiller and Fung, 2007).

Another important pharmacophore group is the morpholine nucleus incorporated in a wide variety of therapeutically important drugs, one of which is linezolid which belongs to the oxazolidinone class of antibiotics and is used for the treatment of infections caused by gram-positive bacteria (Wyrzykiewicz *et al*., 2006; Dixit *et al*., 2005; Raparti *et al*., 2009; Bektas *et al*., 2010, 2012; Bayrak *et al*., 2009a, b). In addition, 4-phenylmorpholine derivatives have been reported to possess antimicrobial, anti-inflammatory, and central nervous system activities (Dixit *et al*., 2006), Oxazolidinones are a relatively new class of synthetic antibacterial agents, having a new mechanism of action that involves early inhibition of bacterial protein synthesis. This class of compounds is particularly active against gram-positive organisms. Oxazolidinones are thought not to be cross-resistant with other types of antibiotics because of their different action mechanisms, which include interaction with the bacterial ribosome to inhibit bacteria. (Zheng *et al*., 2010; Giera *et al*., 2006; Das *et al*., 2005; Gage *et al*., 2000; Cui *et al*., 2005). Hence, oxazolidinone class of antibacterial compounds attracted considerable attention of a number of research groups during the last decade to get more efficacious and less toxic drug (Srivastava *et al*., 2008).

Thiazolidinone derivatives have been further reported to possess diverse pharmacological properties, such as antibacterial, antifungal, anticonvulsant, anticancer, antituberculosis, and antihuman immunodeficiency virus type 1 (HIV-1) activities. Thiazolidinones are novel inhibitors of the bacterial enzyme MurB, a precursor acting during the biosynthesis of peptidoglycan as an essential component of the cell wall of both gram-positive and gram-negative bacteria. (Bonde and Gaikwad, 2004; Aridoss *et al*., 2007; Küçükgüzel *et al*., 2002; Capan *et al*., 1999; Barreca *et al*., 2001; Andres *et al*., 2000; El-Gaby *et al*., 2009)

The identification and synthesis of combinational chemotherapeutic drugs with different mechanisms of action and with few side effects are an important part of the efforts to overcome antimicrobial resistance (Bayrak *et al*., 2010a, b). A recent survey of novel small-molecule therapeutics has revealed that the majority of the drugs results from an analog-based approach and that their market share represents two-thirds of all drug sales (Vicini *et al*., 2008).

In the present study, as a part of our ongoing study on the synthesis of bioactive hybrid molecules, we aimed to obtain the far derivatives of linezolid. It was reported that SAR studies of linezolid demonstrated a high tolerance for structural variation at the 4-position of the phenyl ring (Weidner-Wells *et al*., 2002). In the structures of the newly synthesized compounds, the phenyl ring substituted by pyridine and oxazolidinone scaffold by other azole rings such as 1,3-thiazole, 1,3-thiazolidinone, 1,2,4-triazole, 1,3,4-thiadiazole, and 1,3,4-oxadiazole nucleus.

## Results and discussion

**Scheme 1:**
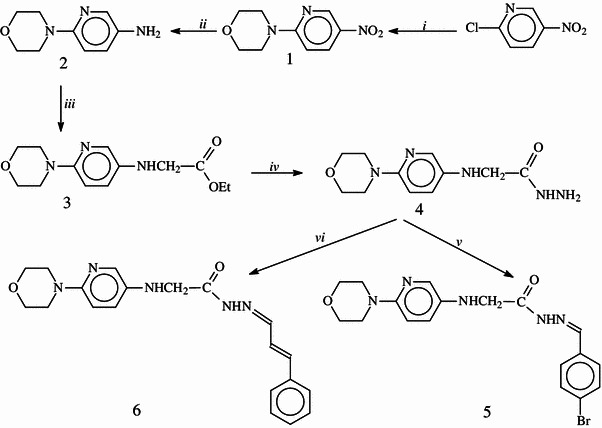
Synthetic pathway for the preparation of compounds **1**–**6**. *i* morpholine, *ii* Pd/C catalyst, H_2_NNH_2_, *iii* BrCH_2_CO_2_Et, *iv* H_2_NNH_2_, *v* BrC_6_H_4_CHO, *vi* C_6_H_5_CH=CHCHO

**Scheme 2:**
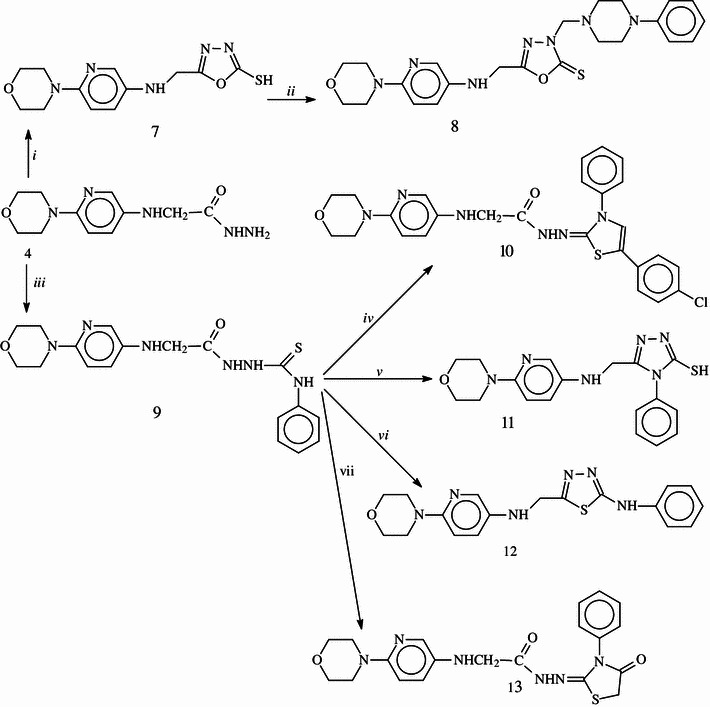
Synthetic pathway for the preparation of compounds **7**–**13**. *i* CS_2_/KOH, *ii* phenyl piperazine, *iii* PhNCS, *iv* BrCH_2_COC_6_H_4_(4-), *v* NaOH, *vi* H_2_SO_4_, *vii* BrCH_2_CO_2_Et

The synthetic route for the newly synthesized compounds (**3**–**13**) is illustrated and outlined in Schemes 1 and 2.

The synthesis of compound **3** was performed from the reaction of ethyl bromoacetate with compound **2** that is available commercially. Then, compound **3** was converted to the corresponding hydrazide (**4**) by the treatment with hydrazine hydrate. The FT-IR and ^1^H NMR spectra of compound **4** displayed signals pointing the presence of hydrazide function, whereas the signals due to ester group disappeared in the NMR spectrum. The treatment of hydrazide, **4** with aromatic aldehydes, namely, 4-bromobenzaldehyde and cinnamaldehyde produced the corresponding Schiff bases, compounds **5** and **6**. In the ^1^H NMR spectra of these compounds, the signal derived from NH_2_ group disappeared; instead, new signals originated from aldehyde moiety were recorded at the related chemical shift values in the ^1^H NMR and ^13^C NMR spectra. Moreover, these compounds (**5** and **6**) exhibited EI-MS and elemental analysis data consistent with the proposed structures.

The synthesis of 5-{[(6-morpholin-4-ylpyridin-3-yl)amino]methyl}-1,3,4-oxadiazole-2-thiol (**7**) was carried out from the reaction of hydrazide **4** with carbon disulfide in the presence of potassium hydroxide. An evidence for the formation of **7** is the absence of the signals corresponding to hydrazide function in the FT-IR and ^1^H NMR spectra. The D_2_O exchangeable signal observed at 13.45 ppm was attributed to the SH proton located at the position-2 of 1,3,4-oxadiazole ring. The reaction of **7** with phenylpiperazine in the presence of formaldehyde afforded the corresponding Mannich base, 5-{[(6-morpholin-4-ylpyridin-3-yl) amino]methyl}-3-[(4-phenylpiperazin-1-yl)methyl]-1,3,4-oxadiazole-2(3*H*)-thione (**8**). In NMR spectra of **7**, the presence of the peaks belonging to 4-phenylpiperazine nucleus confirmed the Mannich reaction.

The synthesis of *N*′-[(5-(4-chlorophenyl)-3-phenyl-1,3-thiazol-2(3*H*)-ylidene]-2-{(6-morpholin-4-ylpyridin-3-yl)amino}acetohydrazide (10) obtained from the cyclocondenzation reaction between 4-chlorophenacyl bromide and compound **9** that was obtained by the treatment of hydrazide 4 with phenylisothiocyanate. On the other hand, the treatment of the same intermediate **9** with ethyl bromoacetate resulted in the formation of 2-[(6-morpholin-4-ylpyridin-3-yl)amino]-*N*′-(4-oxo-3-phenyl-1,3-thiazolidin-2-ylidene)acetohydrazide **13**. The structures of these compounds were confirmed on the basis of FT-IR, EI-MS, ^1^H NMR, ^13^C NMR spectroscopic methods, and elemental analysis.

The basic treatment of intermediate **9** afforded 5-[(6-morpholin-4-ylpyridin-3-yl)methyl]-4-phenyl-4*H*-1,2,4-triazole-3-thiol (11), while the cyclization of **9** in acidic media yielded 5-[(6-morpholin-4-ylpyridin-3-yl)methyl]-*N*-phenyl-1,3,4-thiadiazol-2-amine (**12**). In the ^1^H NMR spectrum of compound **11**, the signal due to SH group was recorded at 13.91 ppm as an evidence of intramolecular cyclization. This group was seen at 2,857 cm^−1^ in the FT-IR spectrum of compound **11**. Two NH signals were recorded at 6.04 and 10.23 ppm as D_2_O exchangeable peaks in the ^1^H NMR spectrum of compound **12**. In the ^13^C NMR spectra of compounds **11** and **12**, other signals belonging to 1,2,4-triazole or 1,3,4-thiadiazole nuclei resonated at the chemical shift values consistent with the literature (Bektas *et al*., 2010, 2012). Furthermore, [M]^+^ ion peaks were observed at the related *m/z* values supporting the proposed structures. In addition, these compounds gave reasonable elemental analysis data.

The newly synthesized compounds **3**–**13** were evaluated in vitro for their antimicrobial activities. The results are presented in the Table 1. Among the compounds tested, compound **8**, which contains different heterocyclic moieties such as morpholine, pyridine, piperazine, and 1,3,4-oxadiazole important antimicrobial activity, was found to be active against all the microorganisms. All compounds except compounds **6**, **7**, **10**, and **13** exhibited activity toward *Mycobacterium smegmatis* (Ms), a nonpigmented, rapidly growing mycobacterium and an atypical tuberculosis factor leading to morbidity and mortality. The highest Ms activity with the MICvalue 15.6 μg/mL was observed for compound **12** that is a 1,2,4-triazole derivative containing morpholine and pyridine nuclei as well. All the tested compounds were found to be active on yeast like fungi, *Candida albicans* (Ca) and *Saccharomyces cerevisiae* (Sc), in high concentrations with the MIC values of 500 or 1,000 μg/mL, whereas all compounds, except compound **8**, displayed no activity against gram-negative bacterial strain. In contrast to other compounds, compound **12** demonstrated a low activity against *Pseudomonas aeruginosa* (Pa), a gram-negative bacillus.

**Table 1:** Antimicrobial activity of the compounds (μg/mL)

Comp. no	Microorganisms and minimal inhibition concentration								
	Ec	Yp	Pa	Ef	Sa	Bc	Ms	Ca	Sc
3	–	–	–	–	–	–	125	1,000	1,000
4	–	–	–	–	–	–	125	500	1,000
5	–	–	–	–	–	–	31.3	1,000	1,000
6	–	–	–	–	–	–	–	500	1,000
7	–	–	–	–	–	–	–	500	1,000
8	62.5	62.5	62.5	31.3	31.3	62.5	125	1,000	1,000
9	–	–	–	–	–	–	125	1,000	1,000
10	–	–	–	–	–	–	–	500	1,000
11	–	–	–	–	–	–	125	500	1,000
12	–	–	500	–	–	–	15.6	500	1,000
13	–	–	–	–	–	–	–	500	1,000
Amp.	8	32	>128	2	2	<1			
Str.							4		
Flu.								<8	<8

Ec*: Escherichia coli* ATCC 25922, Yp: *Yersinia pseudotuberculosis* ATCC 911, Pa: *Pseudomonas aeruginosa* ATCC 43288, Ef: *Enterococcus faecalis* ATCC 29212, Sa: *Staphylococcus aureus* ATCC 25923, Bc: *Bacillus cereus* 702 Roma, Ms: *Mycobacterium smegmatis* ATCC 607, Ca: *Candida albicans* ATCC 60193, Sc: *S. cerevisiae* RSKK 251, Amp.: Ampicillin, Str.: Streptomisin, Flu.: Fluconazole

Almost all the compounds showed moderate-to-good urease inhibitory activity (Table 2). The inhibition was increased with increasing compound concentration. Potent compound have their activities in the range of 2.37–13.23 μM. Lower IC_50_ values indicate higher enzyme inhibitor activity. Compound **10** proved to be the most potent showing an enzyme inhibition activity with an IC_50_ = 2.37 ± 0.19 μM. The least active compound **3** had an IC_50_ = 13.23 ± 2.25 μM.

**Table 2:** The urease inhibitory activity of different concentrations of morpholin derivatives

Compounds	IC_50_ (μM)^a^
**3**	13.23 ± 2.25
**4**	7.92 ± 1.43
**5**	6.87 ± 0.06
**6**	8.29 ± 2.30
**7**	7.01 ± 0.68
**8**	4.99 ± 0.59
**9**	8.07 ± 1.25
**10**	2.37 ± 0.19
**11**	4.77 ± 0.92
**12**	6.05 ± 1.19
**13**	4.46 ± 0.22

^a^Mean ± SD

## Conclusion

In this study, the synthesis of some morpholine derivatives (**3**–**13**) were performed, some of which contain an azole moiety, and their structures were confirmed by IR, ^1^H NMR, ^13^C NMR, Mass spectroscopic, and elemental analysis techniques. In addition, the newly synthesized compounds were screened for their antimicrobial and antiurease activities. Some of them were found to possess activity on *M. smegmatis*, *C. albicans* ATCC, and *S. cerevisiae.* Furthermore, all the compounds exhibited moderate-to-good antiurease activity

## Experimental

### Chemistry

#### General information for chemicals

All the chemicals were purchased from Fluka Chemie AG Buchs (Switzerland) and used without further purification. Melting points of the synthesized compounds were determined in open capillaries on a Büchi B-540 melting point apparatus and are uncorrected. Reactions were monitored by thin-layer chromatography (TLC) on silica gel 60 F254 aluminum sheets. The mobile phase was ethanol:ethyl acetate, 1:1, and detection was made using UV light. FT-IR spectra were recorded as potassium bromide pellets using a *Perkine Elmer* 1600 series FTIR spectrometer. ^1^H NMR and ^13^C NMR spectra were registered on DMSO-*d*_6_ on a *BRUKER AVENE II* 400 MHz NMR Spectrometer (400.13 MHz for ^1^H and 100.62 MHz for ^13^C). The chemical shifts are given in ppm relative to Me_4_Si as an internal reference; *J* values are given in Hz. The elemental analysis was performed on *a Costech Elemental Combustion System* CHNS-O elemental analyzer. All the compounds gave C, H, and N analysis results within ±0.4 % of the theoretical values. The mass spectra were obtained on a *Quattro LC*–*MS* (70 eV) Instrument. Compounds **1** and **2** are available commercially.

### Synthesis of compound **3**

Ethylbromoacetate (10 mmol) was added to the mixture of compound **2** (10 mmol), and triethylamine (10 mmol) was added dropwise in dry tetrahydrofurane at 0–5 °C. Then, the reaction content was allowed to reach to room temperature and stirred for 11 h (the progress of the reaction was monitored by TLC). The precipitated triethylammonium salt was removed by filtration. After evaporating the solvent under reduced pressure, a brown solid appeared. This crude product was recrystallized from ethanol–water (1:2) to afford the desired product.

#### *Ethyl N*-*(6*-*morpholin*-*4*-*ylpyridin*-*3*-*yl)glycinate* (**2**)

Yield (1.27 g, 50 %); m.p. 83–84 °C; IR (KBr, ν, cm^−1^): 3,378 (NH), 1,725 (C=O), 1,575 (C=N), 1,118 (C–O); ^1^H NMR (DMSO-*d*_6_, δ ppm): 1.17 (t, 3H, CH_3_, *J* = 7.4 Hz), 3.18 (t, 4H, 2NCH_2_, *J* = 4.8 Hz), 3.69 (t, 4H, 2OCH_2_, *J* = 4.4 Hz), 3.84 (d, 2H, NHCH_2_, *J* = 6.4 Hz), 4.08 (q, 2H, OCH_2_CH_3_, *J* = 7 Hz), 5.57 (t, 1H, NH, *J* = 6.8 Hz), 6.67 (d, 1H, arH, *J* = 9 Hz), 6.92–6.98 (m, 1H, arH), 7.56 (d, 1H, arH, *J* = 2.4 Hz); ^13^C NMR (DMSO-*d*_6_, δ ppm): 14.83 (CH_3_), 45.84 (NHCH_2_), 47.40 (2NCH_2_), 60.94 (CH_2_OCH_3_), 66.74 (2OCH_2_), arC: [108.94 (CH), 123.74 (CH), 132.35 (CH), 138.22 (C), 153.34 (C)], 172.08 (C=O); LC–MS: *m/z* (%) 266.257 [M+1]^+^ (85), 164.12 (94); Anal.calcd (%) for C_13_H_19_N_3_O_3_ : C, 58.85; H, 7.22; N, 15.84. Found: C, 58.65; H, 7.28; N, 15.85.

### Synthesis of compound **4**

Hydrazide hydrate (25 mmol) was added to the solution of compound **2** (10 mmol) in absolute ethanol, and the mixture was allowed to reflux for 7 h. On cooling the reaction mixture to room temperature, a white solid appeared. The crude product was filtered off and recrystallized from ethanol to give the desired compound **4**.

#### *2*-*[6*-*(Morpholin*-*4*-*yl)pyridin*-*3*-*ylamino]acetohydrazide* (**4**)

Yield (2.23 g, 89 %); m.p. 175–177 °C; IR (KBr, ν, cm^−1^): 3341, 3301, 3189 (NH_2_+NH), 1,658 (C=O), 1,578 (C=N), 1,118 (C–O); ^1^H NMR (DMSO-*d*_6_, δ ppm): 3.14 (t, 4H, N–2CH_2_, *J* = 4.8 Hz), 3.77 (t, 4H, O–2CH_2_, *J* = 4.8 Hz), 4.00 (d, 2H, N–CH_2_, *J* = 6.4 Hz), 4.22 (s, 2H, NH_2_), 5.42 (s, 1H, NH), 5.57 (t, 1H, NH, *J* = 6.8 Hz), 6.65 (d, 1H, arH, *J* = 8.4 Hz), 6.94 (m, 1H, arH), 7.56 (s, 1H, arH); ^13^C NMR (DMSO-*d*_6_, δ ppm): 45.22 (CH_2_), 47.42 (N–2CH_2_), 66.73 (O–2CH_2_), arC: [108.99 (CH), 123.83 (CH), 132.36 (CH), 138.70 (C), 151.71 (C)], 172.20 (C=O); LC–MS: *m/z* (%) 252.29 [M+1]^+^ (80), 164.12 (90); Anal.calcd (%) for C_11_H_17_N_5_O_2_ : C, 52.58; H, 6.82; N, 27.87. Found: C, 52.55; H, 6.68; N, 27.95.

### Syntheses of compounds **5** and **6**

The solution of compound **4** (10 mmol) in absolute ethanol was refluxed with appropriate aldehyde (10 mmol) for 6 h. Then, the reaction content was allowed to cool to room temperature, and a solid appeared. This crude product was filtered off and recrystallized from ethanol to obtain the desired compound.

#### *N*-*(4*-*Bromobenzylidene)*-*2*-*[6*-*(morpholin*-*4*-*yl)pyridin*-*3*-*ylamino]acetohydrazide* (**5**)

Yield (3.43 g, 82 %); m.p. 163–164 °C; IR (KBr, ν, cm^−1^): 3,307 (2NH), 1,687 (C=O), 1,590 (C=N), 1,121 (C–O); ^1^H NMR (DMSO-*d*_6_, δ ppm): 3.20 (brs, 4H, N–2CH_2_), 3.73 (brs, 4H, O–2CH_2_), 4.20 (brs, 2H, CH_2_), 6.73 (d, 1H, arH, *J* = 8.6 Hz), 6.99–7.12 (m, 1H, NH), 7.60 (d, 6H, arH, *J* = 6.2 Hz), 8.91 (s, 1H, N=CH), 11.58 (s, 1H, NH); ^13^C NMR (DMSO-*d*_6_, δ ppm): 45.93 (CH_2_), 56.72 (N–2CH_2_), 66.61 (O–2CH_2_), arC: [123.20 (C), 124.90 (C), 129.66 (CH), 130.01 (CH), 130.73 (CH), 130.98 (2CH), 132.51 (2CH), 136.25 (C), 138.16 (C)], 132.62 (N=CH), 166.12 (C=O); LC–MS: *m/z* (%) 418.66 [M]^+^ (78), 265.12 (28); Anal.calcd (%) for C_18_H_20_BrN_5_O_2_: C, 51.69; H, 4.82; N, 16.74. Found: C, 51.60; H, 4.75; N, 16.80.

#### *2*-*{[6*-*(Morpholin*-*4*-*yl)pyridin*-*3*-*yl]amino}*-*N*-*(3*-*phenylallylidene)acetohydrazide* (**6**)

Yield (3.18 g, 87 %); m.p. 194–195 °C; IR (KBr, ν, cm^−1^): 3,208 (2NH), 1,666 (C=O), 1,554 (C=N), 1,120 (C–O); ^1^H NMR (DMSO-*d*_6_, δ ppm): 3.19 (brs, 4H, N–2CH_2_), 3.67 (brs, 4H, O–2CH_2_), 4.08 (d, 2H, CH_2_, *J* = 5.2 Hz), 5.46 (s, 1H, CH), 6.69 (d, 1H, CH, *J* = 8.2 Hz), 6.99 (d, 3H, arH+NH, *J* = 3.2 Hz), 7.35 (d, 3H, arH, *J* = 7.4 Hz), 7.61 (brs, 3H, arH), 7.91 (s, 1H, NH), 11.42 (s, 1H, NH); ^13^C NMR (DMSO-*d*_6_, δ ppm): 47.48 (CH_2_), 56.72 (N–2CH_2_), 66.75 (O–2CH_2_), arC: [125.83 (CH), 126.20 (CH), 127.76 (CH), 129.53 (CH), 132.51 (CH), 136.56 (C), 138.42 (CH), 139.62 (CH), 146.75 (CH), 153.22 (C), 167.52 (C)], 108.98 (CH), 123.84 (CH), 149.48 (N=CH), 172.00 (C=O); LC–MS: *m*/*z* (%) 365.66 [M]^+^ (75), 265.46 (56), 165.23 (90); Anal.calcd (%) for C_20_H_23_N_5_O_2_: C, 65.74; H, 6.34; N, 19.16. Found: C, 65.82; H, 6.36; N, 19.22.

### Synthesis of compound **7**

Compound **4** (10 mmol) and CS_2_ (6.0 mL, 10 mol) were added to a solution of KOH (0.56 g, 10 mol) in 50 mL H_2_O and 50 mL ethanol. The reaction mixture was refluxed for 3 h. After evaporating in reduced pressure to dryness, a solid was obtained. This was dissolved in 300 mL H_2_O and acidified with conc. HCl. The precipitate was filtered off, washed with H_2_O, and recrystallized from ethanol to afford the desired compound.

#### *5*-*{[(6*-*Morpholin*-*4*-*ylpyridin*-*3*-*yl)amino]methyl}*-*1,3,4*-*oxadiazole*-*2*-*thiol* (**7**)

Yield (2.08 g, 71 %); m.p. 221–222 °C; IR (KBr, υ, cm^−1^): 3,299 (NH), 3,071 (Ar CH), 1,535 (C=N), 1,118 (C–O); ^1^H NMR (DMSO-*d*_*6*_, δ ppm): 3.20 (s, 4H, N–2CH_2_), 3.67 (s, 4H, O–2CH_2_), 4.35 (brs, 2H, CH_2_), 5.94 (bs, 1H, NH), 6.71 (d, 1H, arH, *J* = 7.4 Hz), 7.04 (d, 1H, arH, *J* = 9 Hz), 7.67 (s, 1H, arH), 13.45 (s, 1H, SH); ^13^C NMR (DMSO-*d*_*6*_, δ ppm): 38.44–41.36 (DMSO-*d*_6_+CH_2_), 47.15 (N–2CH_2_), 66.67 (O–2CH_2_), arC: [109.22 (CH), 124.70 (CH), 132.04 (CH), 137.20 (C), 150.45 (C)], 163.10 (oxadiazole C-2), 178.54 (oxadiazole C-5); LC–MS: *m*/*z* (%) 293.45 [M]^+^ (45), 294.75 [M+1]^+^ (86), 165.23 (35); Anal.calcd (%) for C_12_H_15_N_5_O_2_S: C, 49.13; H, 5.15; N, 23.87, S, 10.93. Found: C, 49.25; H, 5.10; N, 23.90; S, 10.85.

### Synthesis of compound **8**

To the solution of corresponding compound **7** (10 mmol) in dichloromethane, formaldehyde (37 %, 1.55 mL) and phenyl piperazine (10 mmol) were added, and the mixture was stirred at room temperature for 3 h. After removing the solvent under reduced pressure, a solid was obtained. This crude product was treated with water, filtered off, and recrystallized from ethyl acetate/petroleum ether (1:2) to yield the desired compound.

#### *5*-*{[(6*-*Morpholin*-*4*-*ylpyridin*-*3*-*yl)amino]methyl}*-*3*-*[(4*-*phenylpiperazin*-*1*-*yl)methyl]*-*1,3,4*-*oxadiazole*-*2(3H)*-*thione* (**8**)

Yield (3.79 g, 81 %); m.p. 87–88 °C; IR (KBr, υ, cm^−1^): 3,392 (NH), 1,599 (C=N), 1,118 (C–O); ^1^H NMR (DMSO-*d*_*6*_, δ ppm): 3.14 (s, 4H, N–2CH2), 3.79 (s, 4H, O–2CH2), 4.51 (brs, 2H, CH2), 4.86 (bs, 8H, 4CH2), 5.01 (s, 2H, CH2), 5.43 (bs, 1H, NH), 6.61 (m, 1H, arH), 6.90 (m, 3H, arH), 7.26 (m, 3H, arH), 8.03 (m, 1H, arH); ^13^C NMR (DMSO-*d*_*6*_, δ ppm): 46.33(N–CH_2_), 46.54 (N–CH_2_), 49.52 (N–2CH_2_), 50.16 (N–CH_2_), 50.59 (N–CH_2_), 66.97 (O–2CH_2_), 70.28 (2CH_2_), arC: [107.98 (CH), 116.64 (2CH), 117.32 (CH), 120.39 (CH), 129.43 (2CH), 133.42 (C), 136.29 (CH), 151.39 (C), 156.61 (C)], 173.47 (oxadiazole C-2), 178.99 (oxadiazole C-5); LC–MS: *m*/*z* (%) 466.85 [M]^+^ (54), 468.11 [M+1]^+^ (36), 215.45(55); Anal.calcd (%) for C23H29N7O2S: C, 59.08; H, 6.25; N, 20.97, S, 6.86. Found: C, 59.18; H, 6.20; N, 20.82; S, 6.88.

### Synthesis of compound **9**

The mixture of compound **4** (10 mmol) and phenylisothiocyanate (10 mmol) in absolute ethanol was refluxed for 6 h. On allowing the reaction content to be cooled to room temperature, a white solid was formed. This crude product was filtered off and recrystallized from ethylacetate to afford the desired compound.

#### *2*-*{[(6*-*Morpholin*-*4*-*ylpyridin*-*3*-*yl)amino]acetyl}*-*N*-*phenylhydrazinecarbothioamide* (**9**)

Yield (3.16 g, 82 %); m.p. 171–172 °C; IR (KBr, ν, cm^−1^): 3,321 (2NH), 3,164 (2NH), 1,685 (C=O), 1,215 (C=S), 1,110 (C–O); ^1^H NMR (DMSO-*d*_6_, δ ppm): 3.02 (bs, 4H, N–2CH_2_), 3.58 (bs, 4H, O–2CH_2_), 3.82 (d, 2H, CH_2_, *J* = 5.2 Hz), 5.85 (s, 1H, NH), 6.42–6.52 (m, 2H, arH), 6.92 (d, 2H, arH, *J* = 9.8 Hz), 7.26 (d, 2H, arH, *J* = 9.4 Hz), 7.75 (bs, 2H, arH), 9.55 (s, 1H, NH), 9.72 (bs, 1H, NH), 10.42 (s, 1H, NH); ^13^C NMR (DMSO-*d*_6_, δ ppm): 45.32 (CH_2_), 55.54 (N–2CH_2_), 66.35 (O–2CH_2_), arC: [101.52 (CH), 114.56 (CH), 125.83 (CH), 126.20 (CH), 128.24 (CH), 132.51 (CH), 136.56 (C), 138.42 (CH), 139.62 (CH), 146.75 (C), 153.22 (C)], 170.56 (C=O), 182.23 (C=S); LC–MS: *m*/*z* (%) 386.25 [M]^+^ (68), 265.24 (66), 165.85 (87); Anal.calcd (%) for C_18_H_22_N_6_O_2_S: C, 55.94; H, 5.74; N, 21.75; S, 8.30. Found: C, 55.82; H, 5.82; N, 21.62; S, 8.42.

### Synthesis of compound **10**

4-Chlorophenacylbromide (10 mmol) and dried sodium acetate (16.4 g 200 mmol) was added to the solution of compound **9** in absolute ethanol, and the reaction mixture was refluxed for 7 h. Then, the mixture was cooled to room temperature, poured into ice-cold water under stirring, and left overnight in cold. The formed solid was filtered, washed with water three times and recrystallized from ethanol to afford compound **10**.

### *N′*-*[(5*-*(4*-*Chlorophenyl)*-*3*-*phenyl*-*1,3*-*thiazol*-*2(3H)*-*ylidene]*-*2*-*{(6*-*morpholin*-*4*-*ylpyridin*-*3*-*yl)amino}acetohydrazide* (**10**)

Yield (3.33 g, 64 %); m.p. 168–169 °C; IR (KBr, ν, cm^−1^): 3,283 (2NH), 1,699 (C=O), 1,588 (C=N), 1,116 (C–O); ^1^H NMR (DMSO-*d*_6_, δ ppm): 3.34 (bs, 4H, N–2CH_2_), 3.81 (d, 4H, O–2CH_2_, *J* = 4.8 Hz), 4.87 (s, 2H, CH_2_), 5.65 (s, 1H, NH), 6.57 (d, 1H, CH, *J* = 8.6 Hz), 7.31 (m, 3H, arH), 7.44–7.57 (m, 6H, arH), 7.97 (d, 3H, arH, *J* = 8.6 Hz), 10.54 (s, 1H, NH); ^13^C NMR (DMSO-*d*_6_, δ ppm): 41.19 (CH_2_), 47.15 (N–2CH_2_), 66.99 (O–2CH_2_), arC: [126.99 (2CH), 129.47 (2CH), 130.21 (2CH), 130.57 (2CH), 130.84 (2CH), 135.64 (2C), 134.05 (2CH), 136.24 (2C), 140.82 (C)], 125.83 (CH, tiyazol C-4), 152.30 (tiyazol C-2), 153.84 (tiyazol C-5), 192.20 (C=O); LC–MS: *m*/*z* (%) 521.25 [M]^+^ (45), 215.45 (65), 165.45 (75); Anal.calcd (%) for C_26_H_25_ClN_6_O_2_S: C, 59.94; H, 4.84; N, 16.13, S, 6.15. Found: C, 59.85; H, 4.78; N, 16.22; S, 6.18.

### Synthesis of compound **11**

A solution of compound **9** (10 mmol) in ethanol:water (1:1) was refluxed in the presence of 2N NaOH for 3 h, then, the resulting solution was cooled to room temperature, and acidified to pH 4 with 37 % HCl. The precipitate formed was filtered off, washed with water, and recrystallized from ethyl acetate to afford the desired compound.

#### *5*-*[(6*-*Morpholin*-*4*-*ylpyridin*-*3*-*yl)methyl]*-*4*-*phenyl*-*4H*-*1,2,4*-*triazole*-*3*-*thiol* (**11**)

Yield (3.17 g, 87 %); m.p. 165–166 °C; IR (KBr, ν, cm^−1^): 3,327 (NH), 3,093 (Ar CH), 2,857 (SH), 1,451 (C=N), 1,115 (C–O); ^1^H NMR (DMSO-*d*_6_, δ ppm): 3.17 (s, 4H, N–2CH_2_), 3.66 (s, 4H, O–2CH_2_), 4.06 (d, 2H, CH_2_, *J* = 2.2 Hz), 5.51 (bs, 1H, NH), 6.68 (d, 1H, arH, *J* = 6 Hz), 6.81 (d, 1H, arH, *J* = 4.0 Hz), 7.44 (bs, 2H, arH), 7.52 (bs, 4H, arH), 13.91 (s, 1H, SH); ^13^C NMR (DMSO-*d*_6_, δ ppm): 38.90–41.41 (DMSO-*d*_6_+CH_2_), 47.27 (N–2CH_2_), 66.72 (O–2CH_2_), arC: [108.81 (CH), 124.04 (2CH), 128.74 (2CH), 130.05 (2CH), 132.70 (CH), 134.16 (C), 137.63 (C), 151.06 (C)], 153.48 (triazole C-3), 168.73 (triazole C-5); LC–MS: *m*/*z* (%) 368.22 [M]^+^ (62), 165.45 (80); Anal.calcd (%) for C_18_H_20_N_6_OS: C, 58.68; H, 5.47; N, 22.81, S, 8.70. Found: C, 58.72; H, 5.42; N, 22.80; S, 8.82.

### Synthesis of compound **12**

Concentrated sulfuric acid (64 mmol) was added into compound **9** (10 mmol) drop by drop under stirring, and the reaction content was stirred in an ice bath for 15 min. The mixture was allowed to reach to room temperature and stirred for an additional 3 h. Then, the resulting solution was poured into ice-cold water and made alkaline to pH 8 with ammonia. The precipitated product was filtered, washed with water, and recrystallized from ethanol to afford the desired product.

#### *5*-*[(6*-*Morpholin*-*4*-*ylpyridin*-*3*-*yl)methyl]*-*N*-*phenyl*-*1,3,4*-*thiadiazol*-*2*-*amine* (**12**)

Yield (2.13 g, 58 %); m.p. 172–173 °C; IR (KBr, ν, cm^−1^): 3,252 (2NH), 3,077 (Ar CH), 1,599 (C=N), 1,121 (C–O); ^1^H NMR (DMSO-*d*_6_, δ ppm): 3.49 (bs, 4H, N–2CH_2_), 3.66 (bs, 4H, O–2CH_2_), 4.49 (s, 2H, CH_2_), 6.04 (bs, 1H, NH), 7.26–7.34 (m, 4H, arH), 7.54–7.66 (m, 4H, arH), 10.23 (s,1H, NH); ^13^C NMR (DMSO-*d*_6_, δ ppm): 34.63 (CH_2_), 47.18 (N–2CH_2_), 66.69 (O–2CH_2_), arC: [109.13 (CH), 117.93 (2CH), 122.42 (2CH), 125.33 (CH), 129.75 (2CH), 137.53 (C), 141.31 (C), 153.50 (C)], 161.75 (thiadiazole C-2), 165.11 (thiadiazole C-5); LC–MS: *m*/*z* (%) 368.45 [M]^+^ (56), 165.45 (85); Anal.calcd (%) for C_18_H_20_N_6_OS: C, 58.68; H, 5.47; N, 22.81, S, 8.70. Found: C, 58.74; H, 5.55; N, 22.85; S, 8.75.

### Synthesis of compound **13**

Ethyl bromoacetate was added to the solution of compound **9** in absolute ethanol (10 mmol), and the mixture was refluxed in the presence of dried sodium acetate (16.4 g 200 mmol) for 9 h. Then, the mixture was cooled to room temperature, poured into ice-cold water under stirring, and left overnight in cold. The formed solid was filtered, washed with water three times, and recrystallized from benzene-petroleum ether (1:2) to afford the pure compound.

#### *2*-*[(6*-*Morpholin*-*4*-*ylpyridin*-*3*-*yl)amino]*-*N’*-*(4*-*oxo*-*3*-*phenyl*-*1,3*-*thiazolidin*-*2*-*ylidene)acetohydrazide* (**13**)

Yield (3.33 g, 45 %); m.p. 201–202 °C; IR (KBr, ν, cm^−1^): 3,326 (2NH), 1,746 (2C=O), 1,492 (C=N), 1,119 (C–O); ^1^H NMR (DMSO-*d*_6_, δ ppm): 3.17 (bs, 4H, N–2CH_2_), 3.67 (bs, 4H, O–2CH_2_), 3.86 (d, 2H, CH_2_, *J* = 3.8 Hz), 4.18 (s, 2H, S–CH_2_), 5.74 (bs, 1H, NH), 6.89–7.16 (m, 5H, arH), 7.32–7.38 (m, 3H, arH), 10.86 (s, 1H, NH); ^13^C NMR (DMSO-*d*_6_, δ ppm): 30.61 (NH–CH_2_), 45.58 (thiazolidine-CH_2_), 56.28 (N–2CH_2_), 66.64 (O–2CH_2_), arC: [107.12 (CH), 108.79 (CH), 121.52 (CH), 124.15 (CH), 125.19 (CH), 126.52 (C), 129.52 (CH), 130.02 (CH), 132.84 (CH), 138.32 (C), 148.02 (C)], 152.30 (thiazolidine C-2), 158.39 (thiazolidine C-4), 170.94 (C=O); LC–MS: *m*/*z* (%) 426.52 [M]^+^ (52), 215.86 (64), 165.42 (74); Anal.calcd (%) for C_20_H_22_N_6_O_3_S: C, 56.32; H, 5.20; N, 19.70, S, 7.52. Found: C, 56.42; H, 5.32; N, 19.65; S, 7.62.

### Antimicrobial activity

All test microorganisms were obtained from the Hifzissihha Institute of Refik Saydam (Ankara, Turkey) and were as follows: *Escherichia coli* (*E. coli*) ATCC35218, *Yersinia pseudotuberculosis (Y. pseudotuberculosis*) ATCC911, *Pseudomonas aeruginosa* (*P*. *aeruginosa*) ATCC43288, *Enterococcus faecalis* (*E. faecalis*) ATCC29212, *Staphylococcus aureus* (*S. aureus*) ATCC25923, *Bacillus cereus* (*B. cereus)* 709 Roma, *Mycobacterium smegmatis* (*M. smegmatis*) ATCC607, *Candida albicans* (*C. albicans*) ATCC60193, and *Saccharomyces cerevisiae* (*S. cerevisia*) RSKK 251. All the newly synthesized compounds were weighed and dissolved in hexane to prepare extract stock solution of 20.000 microgram/milliliter (μg/mL).

The antimicrobial effects of the substances were tested quantitatively in respective broth media by means of double microdilution, and the minimal inhibition concentration (MIC) values (μg/mL) were determined. The antibacterial and antifungal assays were performed in Mueller–Hinton broth (MH) (Difco, Detroit, MI) at pH.7.3 and buffered Yeast Nitrogen Base (Difco, Detroit, MI) at pH 7.0, respectively. The micro dilution test plates were incubated for 18–24 h at 35 °C. Brain Heart Infusion broth (BHI) (Difco, Detriot, MI) was used for *M. smegmatis,* and incubated for 48–72 h at 35 °C (Woods *et al*., 2003). Ampicillin (10 μg) and fluconazole (5 μg) were used as standard antibacterial and antifungal drugs, respectively. Dimethylsulfoxide with dilution of 1:10 was used as solvent control. The results are presented in Table 1. Urease inhibitory activity was determined according to Van Slyke and Archibald (Van Slyke and Archibald, 1944), and the results are shown in Table 2.
